# Sub-Periosteal Surgery

**Published:** 1877-07

**Authors:** 


### ARTICLE XI.

### *Sub-periosteal Surgery.*

At the Congress of German Surgeons in Berlin, in April, the President, Professor von Langenback, showed to the Society some specimens of sub-periosteal surgery, which Professor James R. Wood, of Bellevue Hospital, New York, had expressly sent over to the Congress by his house surgeon, Dr. Wiggin. The object of chief interest was an inferior maxilla, which had been completely regenerated after excision. The patient was a young woman, eighteen years old, who suffered from phosphoric necrosis of the lower jaw, the result of working in a match factory. The whole inferior maxilla was affected, and the entire bone was removed sub-periosteally at two separate operations. The patient made a good recovery, and returned to her former occupation, but died three years later, from cerebral abscess. Dr. Wood was fortunate enough to obtain the whole skull. The new maxilla was small, but complete and symmetrical, and must have proved of great benefit to the patient. By the side of the preparation the necrosed jaw which had been removed was displayed. The President stated he had operated in four cases for phosphoric necrosis of the inferior maxilla, and showed specimens and photographs of patients in whom the bone had been reformed. These cases proved that the newly produced jaws remain, although it was an admitted fact that in subperiosteal excision of the superior mixslla there was only slight new osseous formation. He thought that if the whole inferior maxilla was to be taken away, it was better to do so by two operations rather than by a single one, as if the jaw is removed altogether at once, retraction is apt to follow. In conclusion, he thanked Professor Wood for his kindness in sending his specimens to Germany.—*Med. and Surg. Reporter.*

